# Treatment of vocal fold scarring with autologous bone marrow-derived human mesenchymal stromal cells—first phase I/II human clinical study

**DOI:** 10.1186/s13287-020-01632-8

**Published:** 2020-03-20

**Authors:** Stellan Hertegård, Srinivasa Rau Nagubothu, Emma Malmström, Katarina LeBlanc

**Affiliations:** 1 Department of Clinical Sciences and Intervention, Karolinska Institutet, Stockholm, Sweden; 2 Department of Otorhinolaryngology, Karolinska University Hospital Huddinge, S-141 86 Stockholm, Sweden; 3 Department of Laboratory Medicine, Karolinska Institutet, Huddinge, Sweden; 4 Patient Area Cell Therapies and Allogeneic Stem Cell Transplantation, Karolinska University Hospital Huddinge, Stockholm, Sweden

**Keywords:** Vocal fold, Scarring, Hoarseness, Mesenchymal stromal cells, Fibrosis, Immunomodulation, Wound healing

## Abstract

**Background:**

Vocal fold (VF) scarring, caused by surgery or inflammation, often results in severe voice problems or aphonia. Effective lasting treatment is lacking. Previous in vitro and in vivo animal studies reported positive effects on VF scar resolution with mesenchymal stromal cell (MSC) implantation. The principal aim of this study was to examine safety aspects and secondly treatment efficacy vocal fold function in patients with VF scarring and severe voice problems.

**Methods:**

In this open-label phase I/II study, 16 patients were treated with surgical scar resection followed by injection of autologous MSCs (0.5–2 × 10^6^ MSCs/patient). Patients were monitored 1 year for serious adverse events (SAE) or minor complications. Therapeutic efficacy on treated VFs was evaluated by measurement of VF vibrations using high-speed laryngoscopy (HSL) and phonation pressure threshold (PTP) for elasticity and VF function. Patients self-reported voice change using the Voice Handicap Index (VHI).

**Results:**

No SAE or minor side effects were reported. Video ratings of VF vibrations and digitized analysis of HSL and PTP were significantly improved for 62–75% of the patients (depending on parameter). Two patients showed deteriorated VF vibrations, but improved PTP. VHI was significantly improved in 8 patients, with the remaining experiencing no significant change.

**Conclusions:**

The results indicate that local injection of autologous MSC into scarred VFs with severe voice problems may offer a safe and feasible therapeutic option. VF vibration and elasticity were improved in approximately two thirds of treated patients.

This clinical study is registered in clinicaltrials.gov (ID: NCT01981330). Retrospective registration of first patient (20130511). https//: register.clinicaltrials.gov/.

## Background

Voice problems occur in about 9% of the Western population causing communicative and occupational problems or unemployment, resulting in estimated health costs exceeding 11 billion US dollars [1, 2]. Vocal fold (VF) mucosal damage is evident in 60–80% of patients seeking medical help [3]. VF scarring is considered the most common cause of severe voice problem manifesting with severe dysphonia or aphonia, strained phonation, and reduced VF vibrations [4]. Voice therapy for VF scarring is usually ineffective, as well as surgery, which may even worsen the condition [1, 4].

Numerous approaches have been utilized to improve VF function after scarring. Bioimplant injections (for example fat and hyaluronan; HA) to fill out the VF defect and soften the tissue demonstrated some improvement in VF function [5, 6]. Likewise, injection of growth factors such as hepatocyte and basic fibroblast growth factors were examined in in vivo and clinical trials, with positive outcomes [7, 8]. Injection of autologous fibroblast in 5 humans with VD scar showed improved mucosal waves as well as VHI and voice quality [9]. However, currently, there is no long-lasting effective treatment for VF scarring.

Mesenchymal stromal cells (MSC) have anti-inflammatory properties. In vitro studies have demonstrated that MSCs suppress T cell responses, inducing a regulatory phenotype, skewing the innate immune system, and promoting an anti-inflammatory milieu [10, 11]. Adoptive transfer of MSCs, in clinical trials, demonstrated promising results in reversing conditions, such as therapy-refractory graft-versus-host disease and acute respiratory distress syndrome [12, 13]. How MSCs mediate an immunosuppressive function has not been fully elucidated, but appears to include release of paracrine mediators modulating cells within the local environment. Despite low-level engraftment of transplanted MSCs [14], they induce long-term effects within the body via their “hit and run” actions, reducing tissue damage and promoting endogenous healing [11, 15].

Numerous pre-clinical in vivo models to evaluate the effects of local administration of MSCs into scarred VFs have been reported, each suggesting positive effects on wound healing and regeneration of inherent VF characteristics and functionality [16–18]. Our own in vivo model in rabbits demonstrated both short- and long-term effects of MSC injection on VF tissue inflammation, architecture, and function [19–22]. Despite low-level persistence of the MSCs within the injury site, long-term effects were seen on matrix composition and tissue architecture, with lowered type I collagen content, reduced lamina propria (LP) thickening, and normalized histology compared to untreated injured controls [19–22]. Viscoelastic parameters, from rheometry, demonstrated a significant improvement in tissue functionality after MSC treatment [19, 20]. Resection of established VF scar followed by MSC injection gave the same result [21]. Investigation into MSC mode of action within these studies showed that MSCs significantly expedite resolution of acute phase inflammation within the injured tissue (equivalent to scar tissue resection within the clinical context). Acute phase pro-inflammatory cyto/chemokines including interleukin (IL)-1b and IL-8 were reduced within MSC-treated VFs and increased levels of CD163+ anti-inflammatory macrophages within 2–4 days after damage [22].

Our preclinical testing demonstrated the safety of MSC injection into the VF, with no side effects evidenced [18–22]. We have furthermore confirmed the safety and effectiveness of delivering MSCs within HA hydrogel in vivo. These findings provided us with data supporting that HA could be safely used as a delivery vehicle where defects were of a critical size, providing a scaffold for the MSCs [23].

Limited studies have been undertaken in man, with a case study recently reporting positive results 1 year after treating a female patient presenting with VF scarring and hoarseness, with injection of autologous adipose-derived stromal vascular fraction (SVF) [24].

## Methods

### Aim

We aimed with this phase I/II study to evaluate the safety and therapeutic potential for MSC treatment in humans with manifest VF scarring to restore vocal fold function.

### Patients

Ethical permissions (DNR 2010/1650 and DNR 2014/51432) were received from the Stockholm regional ethical review committee. The study design was identical in both permissions with one treatment arm for patients treated with MSC only and another treatment arm where MSC was mixed with a HA gel. The first permission was for the treatment of 8 patients and the second for a continued study including more patients (in total 16, see VF surgery). The study was registered in registration @clinicaltrials.gov (ID: NCT01981330). Patients provided written informed consent before the procedure. The inclusion and non-inclusion criteria are summarized in Table 1. Sixteen patients were included (Table 2). The mean age of the patients was 54 years (30–74 years, 11 males and 5 females). No female patient was pregnant, and all patients were negative for HIV, HBV, HCV, HTLV, syphilis, and lues. Patients were diagnosed using videostroboscopic examination or a high-speed camera by an experienced phoniatrician and later confirmed with direct microlaryngoscopy. All patients had manifest symptoms (≥ 3 years), strained voice, and severe dysphonia. Seven patients had unilateral scar, 9 bilateral, and 5 patients had larger tissue defects (at least 1.5–2 mm glottal closure width defect during phonation). Scarring was caused by previous (> 3 years ago) VF surgery or trauma (*n* = 6), surgery due to laryngeal cancer with (*n* = 2) and without (*n* = 1) radiation therapy (15 years previous). In 6 patients, scarring was combined with sulcus vocalis, and for 1 patient, the etiology was unknown (P5). All patients were previously treated with voice therapy (at least 5–10 sessions) by a speech and language pathologist without improvement. Six patients had been treated with pure HA injections into one VF > 2 years previously, however with no or short-term improvement.

**Table 1:** Inclusion and non-inclusion criteria for the clinical trial: MSC treatment of vocal fold scarring

Inclusion criteria	
Severe hoarseness, vocal fatigue	
Vocal fold scarring	
No active other treatment	
Age above 18 years	
Exclusion criteria	
Active treatment of laryngeal disorder	
Active inflammatory condition of the larynx or laryngeal papilloma	
Diagnosed or suspicions of local malignancy	
No female patient was pregnant and all patients were negative for HIV, HBV, HCV, HTLV, syphilis, and lues

**Table 2:** Summary of Patients data, and Results for Vocal Fold function parameters, Pressure data and Patient’s subjective ratings preoperative and after at 1 year

Patient	Group (vocal fold damage)	Treatment: MSC or MSC+hyaluronan (HA) (unilateral or bilateral)	Side effects: SAE (systemic reaction, airway problem, infection); minor (e.g., fold edema, laryngitis)	Age, sex	Voice Handicap Index (pre/postop 0–120) (*VHI change ≥ 13 points decrease = sign. Improvement)	Vocal fold vibration qualitative ratings (pre/postop): mucosal wave, vibration amplitude, glottal closure	Vocal fold vibration, computerized analysis (pre/postop) (normalized *U*)	Phonation pressure threshold: PTP (cm H^2^O) (pre/postop) **decrease ≥ 0.5 cm = positive change
							Max. area variations (increase = positive), glottal closure (decrease = positive), open/closed coefficient (%) (decrease = positive)	
1	Scar+defect (uni)	MSC, uni	None	66, male	53/24*	Improved	1732/2146, 49/0, 78/68, improved	5.5/4.7**
2	Severe scar (bilat)	MSC, bilat	None	53, male	86/53*	Improved	–	7.8/4.1**
3	Sulcus+scar (bilat)	MSC, uni	None	57, female	78/74	Decreased, Gl. closure improved	1723/2021, 107/40, 82/71, improved	6.4/4.0**
4	Scar+defect (uni)	MSC, uni	None	50, male	65/74	Improved	1432/2124, 76/84, 68/74, unchanged	4.0/2.7**
5	Scar (bilat)	MSC, bilat	None	71, male	93/61*	Improved	1290/1476, 0/0, 75/59, improved	4.3/2.8**
6	Scar (uni) **large defect**	MSC+HA, uni	None	55, male	103/104	Improved	–	5.4/5.5
7	Scar (uni) **large defect**	MSC+HA, uni	None	70, male	59/69	Improved	633/1357, 139/11, 64/78, improved	4.9/5.0
8	Scar (uni) **large defect**	MSC+HA, uni	None	58, male	80/87	Unchanged	1498/3278, 209/540, −, unchanged	4.8/4.4
9	Scar severe (bilat)	MSC, bilat	None	74, male	89/85	Decreased	–, 740/1262,–, decreased	8.3/3.5**
10	Scar (uni) **large defect**	MSC, uni	None	48, female	109/72*	Decreased, Gl. closure improved	1517/2692, 942/553, 58/50, improved	5.7/4.7**
11	Sulcus+scar bilat	MSC+HA, uni	None	42, female	105/51*	Unchanged	2430/3249, 0/0, 88/37, improved	6.6/5.8**
12	Sulcus+scar (bilat)	MSC+HA, uni	None	45, female	113/92*	Improved	1761/1973, 2/0, 79/65, improved	5.9/5.6
13	Sulcus+scar bilat	MSC+HA, uni	None	51, male	74/82	Decreased	3602/4691, 3/59, 76/77, unchanged	6.0/5.3**
14	Scar (uni) **large defect**	MSC+HA, uni	None	48, female	85/35*	Improved	566/1716, 0/0, 51/47, improved	6.5/4.4 **
15	Sulcus+scar (bilat)	MSC, uni	None	43, male	50/13*	Improved	931/1579, 50/0, 75/81, improved	5.7/4.3**
16	Sulcus+scar (bilat)	MSC+HA, uni	None	30, male	86/87	Improved	1216/1210, 439/19, 93/81, improved	8.4/7.6**

Patient 2 had a tracheostomy due to extensive scarring with fixation of cricoarytenoid joints. One patient (no. 9) smoked, and 1 patient suffered several cardiac infarctions the last 2 years previously (no. 5)

### Isolation and characterization of bone marrow MSCs

The MSC expansion procedure was accredited by the Swedish National Board of Health and Welfare (952/2009, 6.3.3-8874/2011, 6.1.3-9791/2013, 6.1.3-16411/201). Autologous MSCs were isolated from the iliac crest for each patient as previously described [25]. Expansion and characterization of MSCs was performed according to guidelines of the European Blood and Marrow Transplantation Group approved by the Swedish National Board of Health and Welfare. Bone marrow mononuclear cells were seeded at a density of 1.6 × 10^5^ cells/cm^2^ in Dulbecco’s modified Eagle’s medium-low glucose supplemented with platelet lysate (final concentration equivalent of 9 × 10^7^ platelets/ml). Platelet concentrate was purchased from the Department of Transfusion Medicine, Karolinska University Hospital, Huddinge, Sweden. At 80–90% confluency, cells were detached with TrypLE™ (Invitrogen, NY, USA) and replated at 3.0–4.0 × 10^3^ cells/cm^2^ for one passage. Cells were cryopreserved in complete cell culture media supplemented with 10% (v/v) dimethyl sulfoxide (DMSO; WAK-Chemie Medical GmbH, Steinbach, Germany). Before use, cells were washed in phosphate-buffered saline and resuspended in 0.9% (v/v) saline solution supplemented with 10% AB Rh+ plasma at a concentration of 2.0 × 10^6^ MSCs/ml. Release criteria were based on the absence of visible clumps, spindle-shaped morphology, absence of contamination by pathogens (bacteria and mycoplasma), and viability > 95%. Flow cytometry confirmed an MSC surface profile as per the International Society for Cellular Therapy guidelines (CD73^+^, CD90^+^, CD105^+^, human leukocyte antigen [HLA]-I^+^ and CD14^−^, CD34^−^, CD3^−^, CD80^−^, CD45^−^ HLA-II^−^) [26]. All patients received MSCs at passage 1.

### Vocal fold surgery and MSC administration

**Fig. 1:**
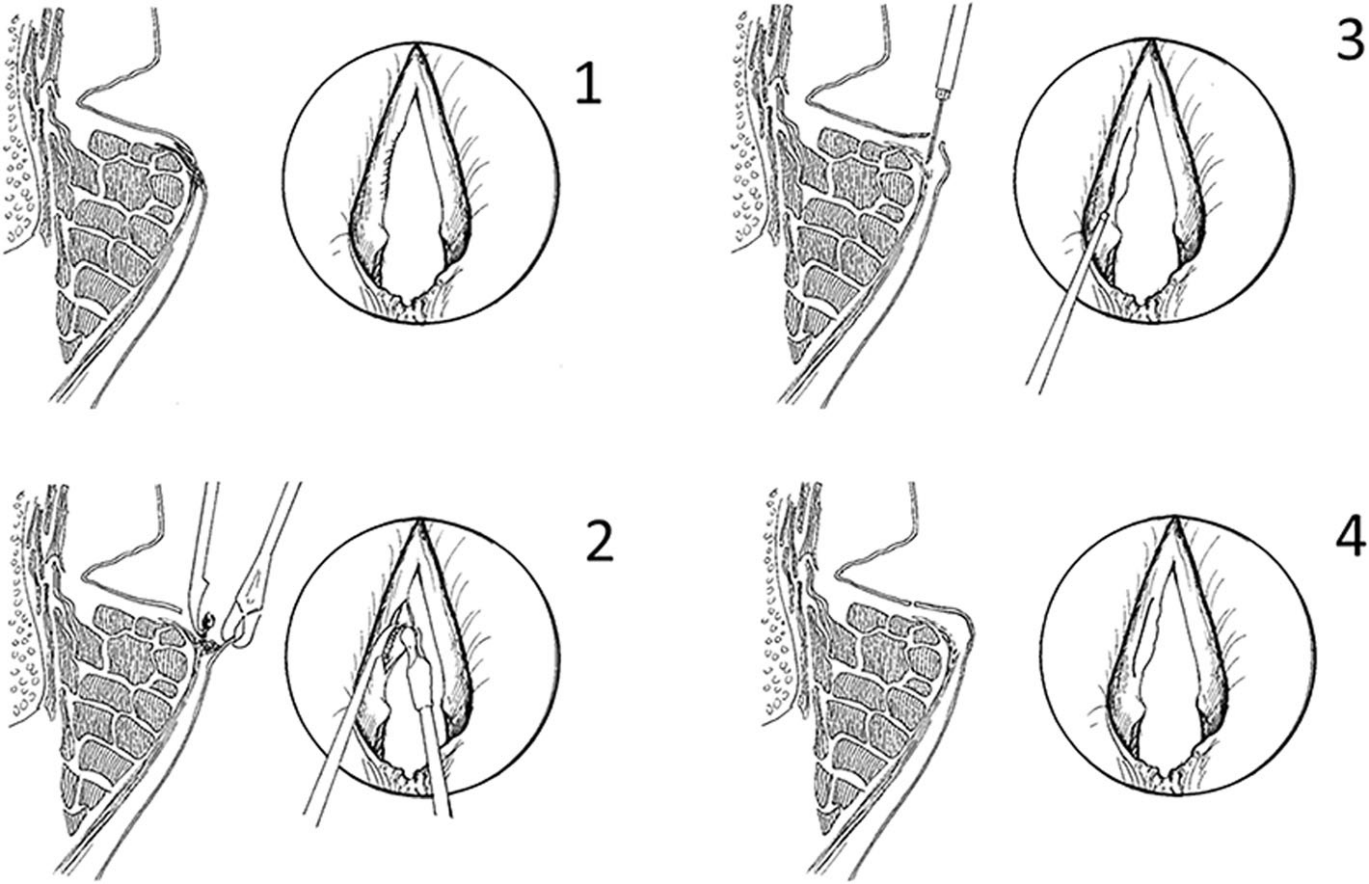
Surgery. Schematic drawing of operative technique. **1** Preoperative status with scar at vocal fold edge. **2** After cordotomy with microflap technique, scar resection. **3** Injection of MSCs in lamina propria and superficial thyroarytenoid muscle (not shown in figure). **4** Directly after surgery

During microlaryngoscopy (Fig. 1), scar tissue was removed/reduced from the LP with minimal epithelium resection to create a fresh wound. Thirteen patients were operated unilaterally on the most scarred and stiffer VF, and 3 patients, where microlaryngoscopy showed severe or symmetrical bilateral scar, were operated bilaterally (P2, P5, P9). MSC injections (0.5–1 × 10^6^ cells/damaged VF [total dosage 0.5–2 × 10^6^/patient dependent on the amount of VF damage and defect size]) were performed using a Medtronic Xomed 27G laryngeal injector into the LP and thyroarytenoid muscle in 8 patients. If leakage was noted at the beginning, the injection was adjusted until a dose of 0.5–1 × 10^6^ cells/damaged VF was administered. No patient was excluded because of leakage. Cell dosage was based on previous animal safety data and adjusted for difference in membranous VF volume between humans and rabbits [19–23]. The ethical permissions also included a second treatment arm where MSC was mixed with a HA gel scaffold. We included 8 randomly chosen patients where the MSCs (cell dosage within the same ranges as above) were mixed with HA gel (Auxigel™; Termira AB, Stockholm, Sweden, [23, 27]). The gel was prepared by mixing 0.9% (w/v) HA in phosphate-buffered saline (PBS; part A) with 0.1% (w/v) polyvinyl alcohol derivative in PBS (to induce crosslinking, part B) at a 3:1 ratio. The aim was to examine if the gel improved cell placement near the wound area and increased healing. All patients were recommended voice rest 5–7 days postoperatively. No antibiotics were given. Five patients declined postoperative voice treatment, with the remaining patients receiving 2–10 sessions. All patients were examined postoperatively at 1 week, 1, 3, 6, and 12 months.

### Side effects and complications

Patients were monitored during and following surgery (between 3 h for day care surgery and 24 h for overnight stay patients) and at each of the follow-up visits. The patients were interviewed and examined for side effects (SAE) including systemic reactions, airway problems, infections, tumor formation, and minor, local effects, e.g., fold edema, laryngitis VF hematoma, and granuloma.

### Analysis of vocal fold vibrations and phonation pressure threshold

Functional vocal fold parameters were analyzed from:

#### High-speed examinations, videostroboscopic recordings

Digitized high-speed recordings were made using a Hispec 1 camera with an image resolution set to 500 × 250 pixels at 4000 images/s (Fastec Imaging, San Diego, USA) combined with a 300 W xenon light source (5131, Richard Wolf GmbH, Knittlingen, Germany). Videostroboscopy was performed with a Wolf stroboscope (5052) attached to a Wolf videocamera (5512). The video was digitized using FonMedia software (Hans Larsson, Karolinska Institutet). A 70° rigid Karl Storz (Tuttlingen, Germany; 8700) laryngoscope or Olympus (ENF-P4) flexible laryngoscope was used for examination. Patients sustained an /ee/ like vowel at different intensities and pitches. The phonation with the best closure, closest to the habitual speaking pitch and intensity, was further analyzed.

#### Subjective video ratings

The recordings were mixed pairwise (pre- and post-operative) randomly adding 10% extra samples for intra-reliability testing of the judges. The judges were 3 experienced phoniatricians, without prior knowledge of the patient’s diagnoses or treatment, who blindly rated the following VF parameters: glottal closure, amplitude of vibration, and mucosal wave. The judges rated the pre- and 1 year post-operative recordings pairwise in random order using the global categories A: best status, B: worse status, and C: unchanged/unclear. Ratings were made for high-speed recordings, except for P2 and P6 where videostroboscopic recordings were used.

#### High-speed computerized analysis

**Fig. 2:**
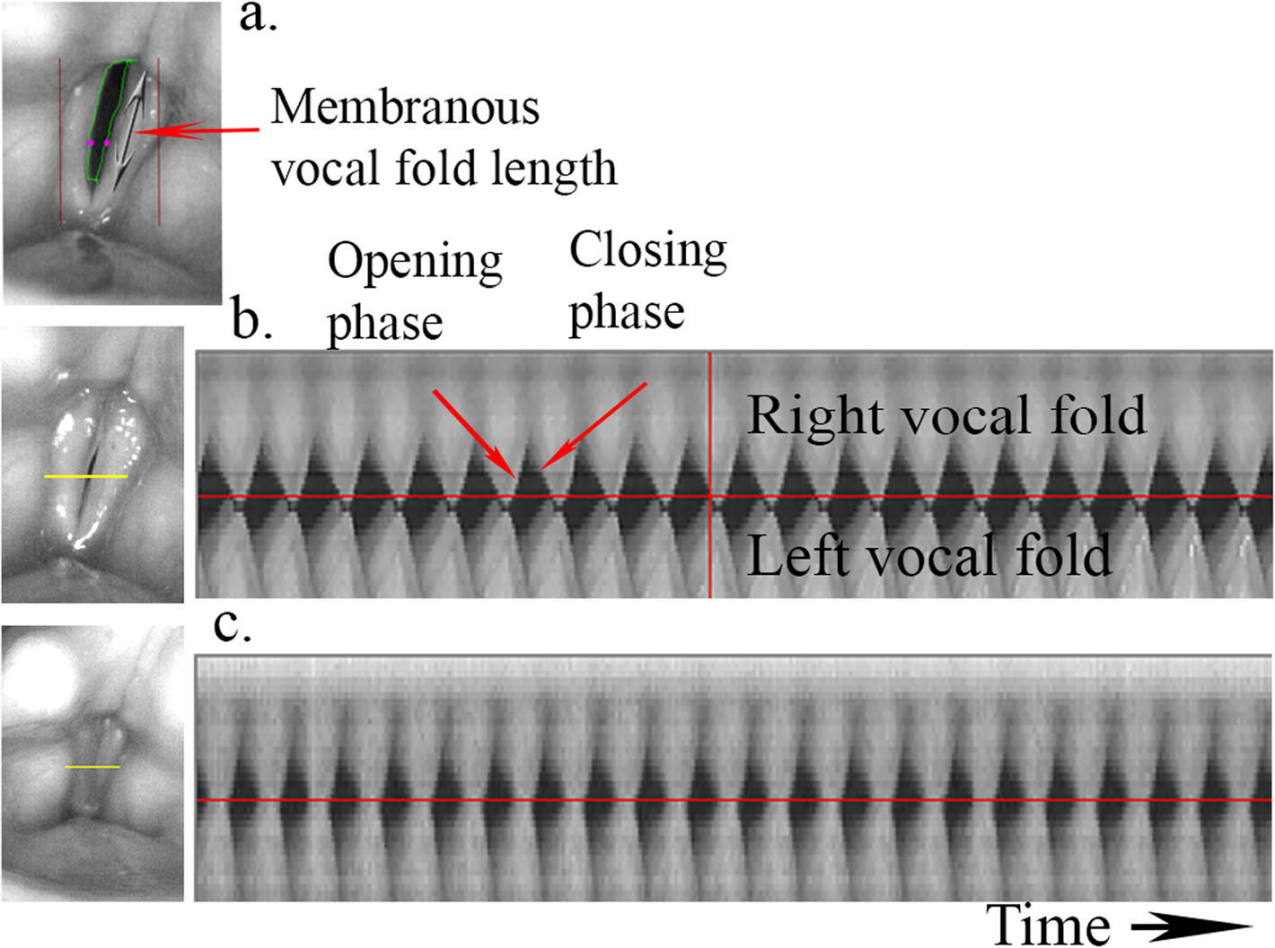
Vocal fold analysis of high-speed laryngoscopy. **a** (Top) Edge tracking of glottal area during vibration. Dots at midpoint of left and right vocal folds. Arrow marks length of membranous vocal fold part use for normalization of vibration and glottal area. **b** (Mid) Preoperative recording. **c** (Bottom) Postoperative recording for patient 12 with corresponding kymograms from the horizontal yellow line plane (left). Right vocal fold vibrations are shown above and left local fold below. Red vertical line at preoperative kymogram corresponds to maximum glottal closure (left image). High-Speed Studio software automatically sets glottal midline (red horizontal) and analyzes the brighter pixels at the most closed phase during each vibratory cycle in relation to the darker pixels during the open phase. Open/closed coefficient is calculated from this relation. Preoperative O/C coefficients in the figure are 73% preoperatively and 52% postoperatively indicating improved glottal closure. Preoperative, there is a time phase delay of maximum closure for the right vocal fold in comparison with the left vocal fold which is normalized after treatment of the right vocal fold

**Fig. 3:**
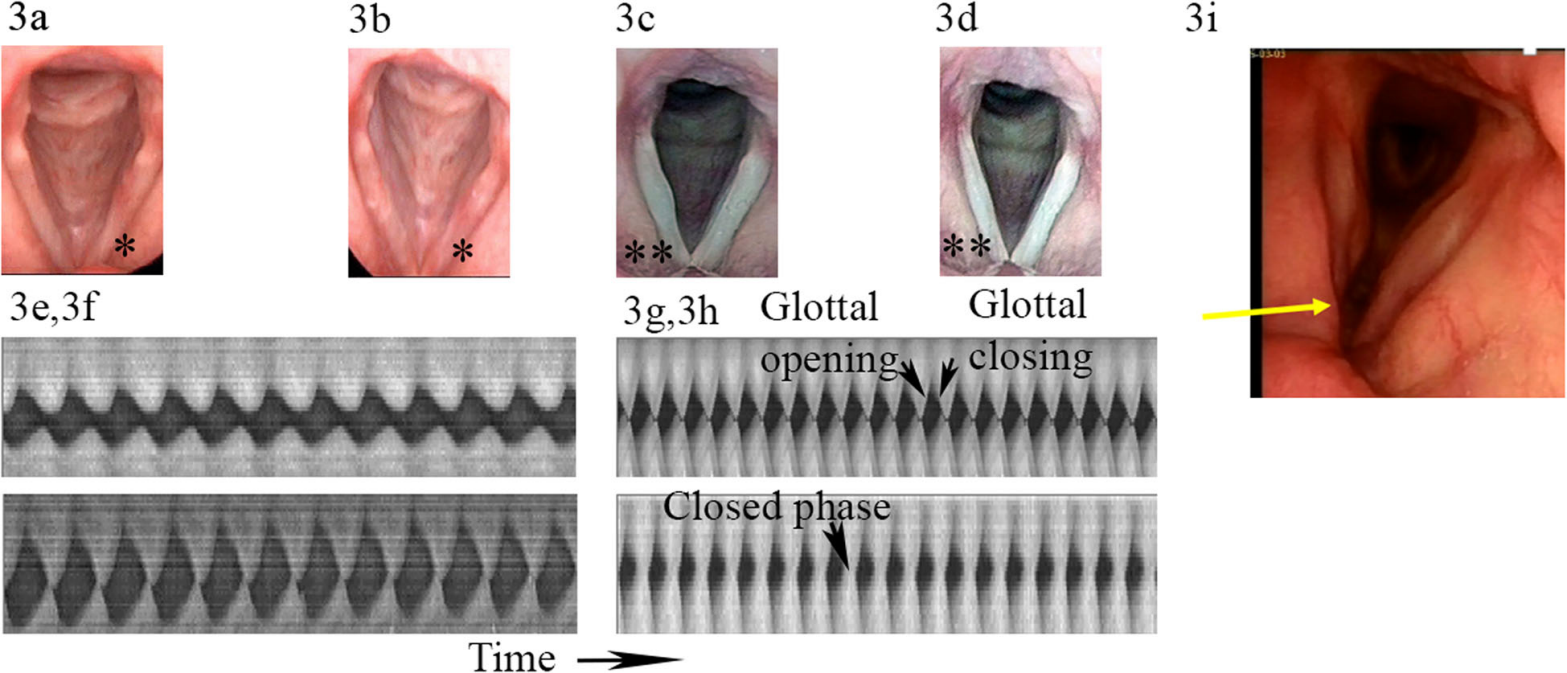
Vocal fold recordings and analysis. (3a, 3b) Patient 1 with small scar defect on left vocal fold preoperative and 1 year postoperative (marked with asterisk). (3c) Patient 12, preoperative a small scar defect on right vocal fold (marked with two asterisks). (3d) Eight months after MSC treatment with restored vocal fold edge. (3e, 3f) Kymograms for patient 1 of vocal fold vibrations preoperative (top) and 12 months postoperative (bottom). Time scale to right. (3e) Incomplete glottal closure and reduced vibrations of the left vocal fold (lower) than right vocal fold (top). (3f) Complete glottal closure at 12 month follow-up. (3g, 3h) Kymograms for patient 12 preoperative with incomplete glottal closure and reduced vibrations (top) and 8 months postoperative with complete closure and more symmetric vocal fold vibrations (bottom). (3i) Patient 6 who has a larger defect anterior on the right vocal fold (arrow)

The high-speed recordings were analyzed using a specially developed software High-Speed Studio (HSS) [28]. Digitized images of glottal area variations during vibration were traced by automatic edge detection and normalized to the membranous VF portion length at the glottal midline (Figs. 2 and 3). Relative glottal area closure (minimum area) and relative maximum glottal area variations (vibrations) were calculated using Sopran (Tolvan Data, Stockholm, Sweden). The open/closed coefficient during vibratory cycles was calculated using HSS from kymograms (Fig. 2). This reflects the degree of glottal closure during phonation [28].

The phonation pressure threshold (PTP) was recorded as a measure of vocal onset effort and indirect estimation of glottal mucosal elasticity [29]. PTP was estimated from intraoral pressure during repeated “pa” syllables at habitual pitch and effort with decreasing intensity until phonation ceased. Pressure (cm H^2^O) was recorded with a 4-mm diameter catheter placed in the corner of the patient’s mouth connected to a log data recorder (Pico Technology, St. Neots UK; model 1012, Pico Scope software, v6). PTP was calculated from a mean of 3 pressure peaks surrounding vowels during stable syllable repetitions at the softest possible phonation.

### Patient’s subjective ratings

The patients rated their voice symptoms using the Voice Handicap Index scale (VHI, Swedish version) including subscales reflecting functional, physical, and emotional aspects of voice [30].

### Statistics

Analysis of patient data was performed using non-parametric methods: Wilcoxon paired sign rank test for pairwise comparisons (pre-operative and 1-year follow-up), Mann-Whitney *U* test for group comparisons of rate of parameter changes between groups, and Binomial test (sign test) for analysis of video ratings. Significance level was set to *p* < 0.05 (Statview 5.0; SAS Institute Inc., Cary, NC USA, Open version).

## Results

### Side effects and complications

No complications or SAEs were reported, e.g., local edema, bleeding, granuloma, tumor formation, or signs of infection during the observation time (Table 2).

### High-speed examination, videostroboscopic, and PTP analyses

The intra- and inter-rater reliability for the qualitative video ratings was satisfactory (over 70% of the judgments fell in the same category for the doubled samples). Video ratings demonstrated improvement in 10 patients, with no evaluable change in further 2 patients. In 4 patients, VF vibrations decreased; however, for 2 individuals, glottal closure was improved, which is also important to voice production (Table 2). Taken together, 10 patients showed improvement, 2 decreased, and 4 unchanged based on video rating. Binomial sign test for 10 patients with improved and 4 with decreased vibrations resulted in *p* = 0.176, and for 10 improved and 2 decreased, a significant improvement was found, *p* = 0.0386 (Tables 2 and 3).

**Table 3:** Statistical evaluation of vocal fold function parameters

Parameter	All patients (*n* = 16)	Patients treated with MSC only (*n* = 8) or MSC+hyaluronan (HA) (*n* = 8)	Patients with smaller defects (*n* = 11)	Patients with large defects (*n* = 5)
VHI (subjective voice handicap scale)	T0, 83 (SD 19); T1, 66 (SD 25) (*p* = 0.04)	MSC: T0,78 (SD 20.5); T1, 57 (SD 25.7) (*p* = 0.036) MSC+HA: T0, 88 (SD 18); T1 76 (SD 23) (*p* = ns)	T0, 81 (SD 20); T1, 63 (SD 26) (*p* = 0.04)	T0, 87 (SD 20); T1,73 (SD 26); ns
Phonation pressure threshold, PTP (cm H^2^O)	T0, 6.0 (SD 1.3); T1, 4.7 (SD 1.2) (*p* = 0.0008)	MSC: T0, 6.0 (SD 1.5); T1, 3.85 (SD 0.8) (*p* = 0.01) MSC+HA: T0, 6.1 (SD 1.15); T1, 5.45 (SD 1.0) (*p* = 0.36)	T0, 6.2 (SD 1.5); T1, 4.6 (SD 1.4) (*p* = 0.003)	T0, 5.5 (SD 0.7); T1, 4.8 (SD 0.5); ns
Maximum amplitude of glottal vibrations (*U*)	T0, 1551 (SD 760); T1, 2270 (SD 982) (*p* = 0.0019)	MSC: T0,1430 (SD 275); T1, 2006 (SD 440) (*p* = 0.03) MSC+HA: T0, 1672 (SD 1068); T1, 2496 (SD 1280) (*p* = 0.03)	T0, 1751 (SD 767); T1, 2274 (SD 1074) (*p* = 0.01)	T0, 1054 (SD 525); T1, 2261 (SD 882); ns (*p* = 0.07)
Open/closed quotient coefficient (%)	T0, 75.4 (11.5); T1, 65.8 (15.3); ns	MSC: T0, 72.7 (SD 8.5); T1, 67.2 (SD 11.1) (*p* = ns) MSC+HA: T0, 75.2 (SD 15.5); T1, 68.8 (SD 20.6) (*p* = ns)	T0, 79.3 (7.5); T1, 68.3 (14.0) (*p* = 0.05)	T0, 57.5 (9.2); T1, 67.5 (23) ns
Glottal vibration parameters ratings (3 judges)	10/16 patients improved, ns; or 12/16, including patients with improved glottal closure (*p* = 0.039)	MSC: 5/8 patients improved, 3/8 decreased (but 2 of these showed improved glottal closure) MSC+HA: 5/8 patients improved, 2/8 were unchanged, and 1/8 decreased	7/11 patients improved; or 8/11, including 1 patient with improved glottal closure	3/5 patients improved; or 4/5, including 1 patient with improved glottal closure

*T0* preoperative, *T1* 1 year follow-up

**Fig. 4:**
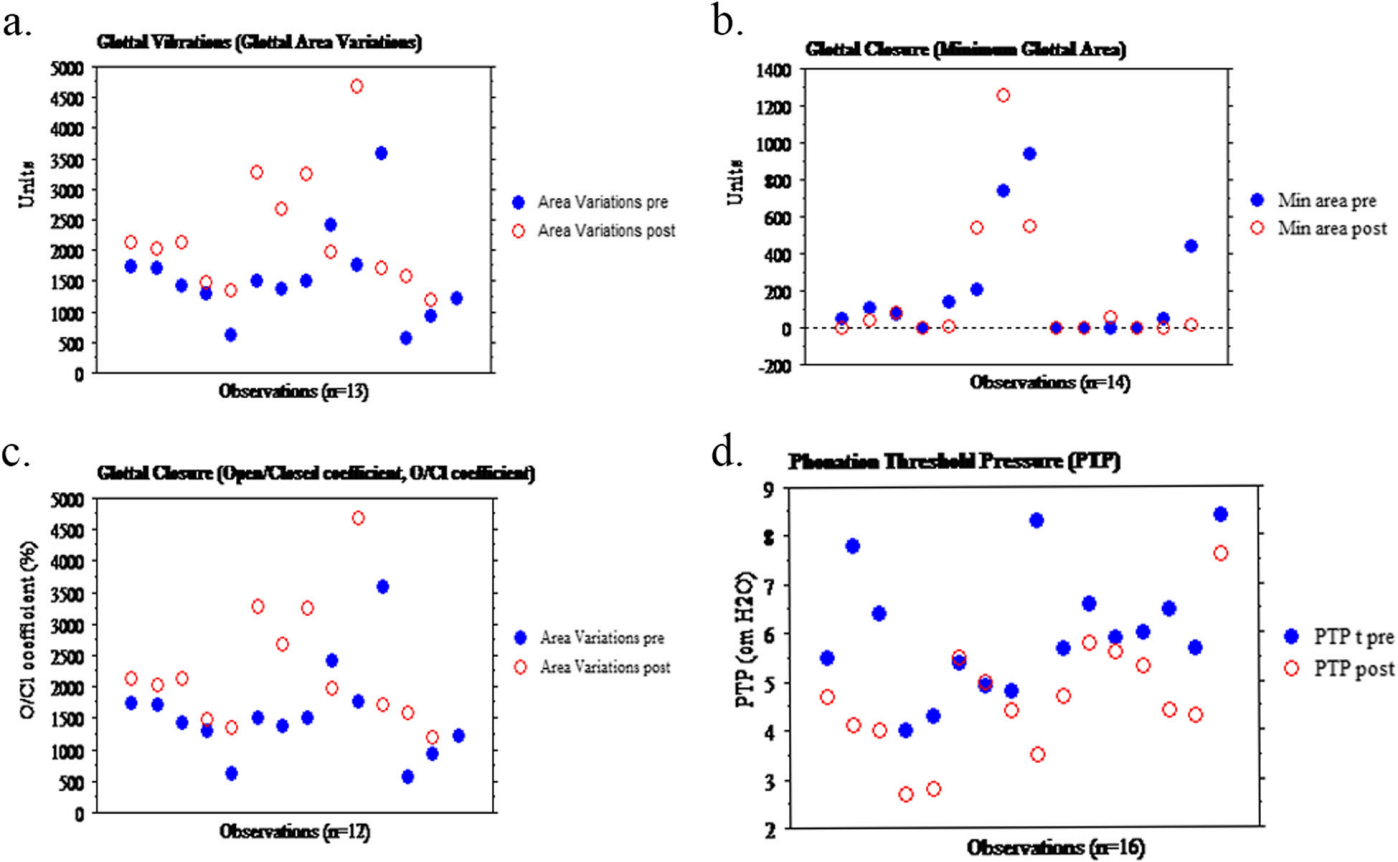
Computerized analysis of vocal fold vibrations and phonation threshold pressure (PTP) results. Results presented as univariate plots pairwise before and 1 year after MSC treatment for each patient (maximum 16 observations before and after treatment). PTP results (d) for all 16 patients. Results for glottal minimum area (b) for 14 patients (2 patients were only examined with videostroboscopy and not with high-speed camera), open/closed coefficient (c) for 12 patients (2 were not examined with high-speed camera and for 2 patients the automatic analysis failed), glottal area variations (a) for 13 patients (2 were not examined with high-speed camera and for 1 patient the automatic analysis failed)

For the computerized analyses, no statistical difference was found for phonation frequency (F0) or sound pressure level (SPL) between preoperative and follow-up examinations. Vocal fold vibration data showed improvement (for at least 2 out of the 3 parameters analyzed) in 10 out of 14 patients, unchanged in 3, and deterioration in 1. The results from computerized analysis of the high-speed recordings and for PTP before and 1 year after treatment are shown in Fig. 4. The pairwise comparisons show a clear improvement for the vibrations (glottal area variations) and for PTP, whereas the glottal closure measurements show a mixed result (minimum glottal area and open/closed coefficient).

The PTP parameter indirectly reflects VF elasticity. We found improvement (decrease ≥ 0.5 cm H^2^O) in 12 patients and no change in 4. Patients 9 and 13 who showed decreased or unchanged vibrations were both improved for the PTP (Table 2 and Fig. 4).

### Patient’s subjective ratings, VHI

VHI ratings (Table 2) showed a clinically significant improvement post-operative (> 13 points improvement) in 8 patients (for all subscales) and for remaining patients no significant change. Two patients rated their voice as normal or close to normal (with 20 points as the cutoff border between normal and deviant voice, 30).

### Statistical analysis and summary of vocal fold function analysis and subjective ratings

Table 3 shows significant improvement for the maximum vibration amplitude, PTP, vibration ratings, and the VHI total score. The results were clearly better for the patients with smaller scar defects as compared to patient with larger defects. Glottal closure (open/closed quotient and closure area Tables 2 and 3) improved after treatment, however not significantly. There was no significant difference in results between the patients who received MSC injections in suspension compared to those injected with MSC+HA gel, except for the PTP which decreased significantly more for the MSC in suspension group (*p* = 0.006). Also, VHI decrease was significant in the MSC-treated group, but not in the MSC+HA group (Table 3). Higher numbers of injected MSCs did not correlate to improved VF parameters or decreased VHI ratings. Maximum vibration amplitude, open/closed quotient, PTP, and VHI improved significantly for the female patients (*p* = 0.04), whereas the male patients improved for vibration amplitude and PTP (*p* = 0.02 and *p* = 0.001 respectively). There was no difference in results between the patients who did receive postoperative voice therapy (*n* = 11) or not (*n* = 5).

## Discussion

To the authors’ knowledge, this is the first phase I/II clinical study documenting use of autologous bone marrow-derived MSC treatment in humans with VF scarring. Here we report no acute or long-term side effects or complications from MSC treatment in the evaluated 1 year after treatment. We have furthermore followed the patients with a standard clinical follow-up of 3–5 years and noted no side effects or complications. An excellent safety profile is in line with results from intravenous (IV) MSC administration [13, 14]. We could not analyze engraftment or survival of the administered MSCs within this trial for ethical reasons, but in animal models, MSC mode of action has been demonstrated to be via a “hit and run” effect, with few cells persisting within the VF tissue over 1 month after injection [18–23].

VF scarring is a condition resulting in severe voice problems for which lasting effective treatment has been elusive. The patients in this study received one single MSC injection with stable results for at least 1 year post-treatment. The most significant improvement was in VF vibration capacity/elasticity. VF vibration parameters were improved for 62–75% of the patients. The majority of the patients experiencing clinical improvement reported that phonation was easier, being able to speak with less effort. This corresponds well to the improvement for the vibration parameters reflecting improved VF elasticity.

For most of the patients, the positive change in glottal parameters and in PTP became evident after 3 months (video files). This indicates an ongoing positive effect from the MSC treatment on healing with less vocal fold stiffness and tissue fibrosis. These clinical findings support our in vivo data outlining the ability of MSCs to exert long-term, indirect effects on the endogenous VF stroma, resulting in improved LP tissue architecture and healing. These findings were evidenced despite the fact that the administered cells were lost from the system within days of delivery [14, 19–23]. We administered MSC one time. It was injected during the VF operation because our previous animal experiments all showed positive effect on VF healing and function if administered in a fresh surgical wound (both in an acute damage and after resecting an established scar in the rabbit VF). Our goal was to mimic this situation. We do not know the optimal time to inject MSC, but most cells die within 24 h after injection in a fresh wound. We believe that early injection is optimal [19–21, 23]. Our previous study also suggests that MSCs shift early wound healing in a non-inflammatory direction [22]. We suggest that per-operative MSC injection or implantation may trigger endogenous healing responses to encourage healing of a more functional tissue repair.


**Additional file 1.** File P1T0. Video file (mp4). Preoperative high speed video file for patient 1. Showing insufficient glottal closure and decreased vocal fold (VF) vibrations on left VF.



**Additional file 2.** File P1T3. Video file (mp4). High speed video file for patient 1 obtained 3 months after treatment. Still insufficient glottal closure and slightly increased vibrations on left VF.



**Additional file 3.** File P1T12. Video file (mp4). High speed video file for patient 1 obtained 12 months after treatment. Glottal closure and clearly increased vibrations on left VF.


Although we could not quantitatively measure the amount of scar, we suggest that the improved VF data indicates less scar tissue after MSC treatment. In 2 of our treated VFs, tissue defects were also restored (e.g., P12 Fig. 3). Any surgery of the VF LP may cause a risk for scarring, and in 4 patients, vibration ratings showed a decrease. However, for 2 of these, the remaining vibration analyses were positive with improvement for glottal closure, computerized vibration data, and PTP. The other 2 patients with decreased vibration ratings both had improved PTP, and patient 13 also improved maximum vibration amplitude, indicating improved VF elasticity. For patient 9, the oldest patient in the study, this could not be measured. The results were less favorable for the 5 patients with scar and larger defects (Table 3). This indicates that MSC injection alone does not seem to regenerate larger defects, which is in line with clinical experience and our previous results after VF resection [19–22].

VHI was significantly improved (> 13 points on total scale) for half of the patients, on all subscales. The remaining patients had mixed results, but no patient rated significant deterioration. As mentioned, only one VF was treated in 13 of the patients, although 9 patients had bilateral scar. The main aim of the study was to evaluate safety of MSC treatment, and the 3 patients operated bilaterally had severe or symmetrical bilateral scar. This is also the reason why we focused on VF vibrations (which can be measured on the treated VF), and no perceptual or acoustic voice analysis was reported in this study. The limited number of patients and lack of control group are limitations of the study, and it is still early to conclude the efficacy to the treatment. We have therefore planned a further study and recently received approvals from the Swedish Medical Product Agency and from the local ethical committee to start a new open phase I/II clinical trial with MSC treatment of patients with VF scarring. In this study, we chose to have the patients as their own controls and no other control group. As mentioned, the main aim of the study was safety evaluation. A control group with patients operated with scar resection only without cell treatment would risk scar healing with voice deterioration or aphonia. Also, our previous animal study where scar was resected and then treated showed increased scar healing with significant deterioration in VF viscoelasticity in the untreated scarred VFs [21].

## Conclusion

In summary, this study showed an excellent safety profile in humans with VF scarring and severe voice problems treated with MSC injection. Vocal fold vibration analyses showed significant improvement in 62–75% of the patients depending on parameter analyzed. Patients with VF scar and larger defects may require alternative treatment, such as cell therapy and a suitable scaffold. Further investigation of efficacy in a larger trial is warranted where limitations with regard to defect size could be addressed to improve clinical outcome.

## Data Availability

The datasets generated and/or analyzed during the current study are not publicly available due [Dataare kept protected following the routines of the Institution at Karolinska Institutet], but are available from the corresponding author on reasonable request.

## Abbreviations

HA: Hyaluronan
HSL: High-speed laryngoscopy
HSS: High-Speed Studio software
LP: Lamina propria
MSC: Mesenchymal stromal cells
PTP: Phonation pressure threshold
VF: Vocal fold
VHI: Voice Handicap Index
